# Links between Climate, Malaria, and Wetlands in the Amazon Basin

**DOI:** 10.3201/eid1504.080822

**Published:** 2009-04

**Authors:** Sarah H. Olson, Ronald Gangnon, Eric Elguero, Laurent Durieux, Jean-François Guégan, Jonathan A. Foley, Jonathan A. Patz

**Affiliations:** University of Wisconsin, Madison, Wisconsin, USA (S.H. Olson, R. Gangnon, J.A. Foley, J.A. Patz); Montpellier University, Montpellier, France (E. Elguero, J.-F. Guégan); Institut de Recherche pour le Développement, Lago Sul, Brazil (L. Durieux); French School of Public Health, Paris, France (J.-F. Guégan)

**Keywords:** Malaria, vector-borne infections, Brazil, precipitation, wetlands, climate, dispatch

## Abstract

Climate changes are altering patterns of temperature and precipitation, potentially affecting regions of malaria transmission. We show that areas of the Amazon Basin with few wetlands show a variable relationship between precipitation and malaria, while areas with extensive wetlands show a negative relationship with malaria incidence.

Global models of malaria can be used to forecast the impact of climate change on malaria, a highly climate-sensitive disease that causes >1 million deaths worldwide each year, mostly in children. However, a limitation of these models is the application of a uniform malaria–precipitation relationship to geographically diverse regions (*1*–*3*). Moreover, the Millennium Ecosystem Assessment has recognized a lack of knowledge about climate-sensitive diseases such as malaria and has called for a “more systematic inventory, by region and country, of current and likely population health impacts of ecosystem change” (*4*). Understanding malaria-precipitation relationships at regional levels will enhance predictability of ecosystem or climate change impact on population health.

Precipitation and surface hydrology are key factors in determining the abundance of *Anopheles* mosquito vectors for malaria. Mosquitoes require pools of water to complete their life cycle, and malaria models have estimated changing transmission by setting minimum levels of precipitation below which mosquito populations are (theoretically) suppressed. However, using a uniform hydrologic threshold for malaria does not capture critical characteristics of landscape, soil, and rainfall (i.e., intensity, frequency), all known contributors to the abundance, persistence, and spatial distribution of mosquito habitats.

In the Amazon Basin, the predominant malaria vector is *Anopheles darlingi*. Short longitudinal studies show that human-landing catches of *An. darlingi*, which breeds along the edges and in debris of clear, partially sunlit pools, are closely associated with local malaria rates (*5*,*6*). These observations establish that biting rates are elevated in regions of elevated malaria risk. Likewise, biting rates correlate with abundance of larvae and larval habitats and proximity of humans to larval habitats (*7*,*8*).

Local observations demonstrate the existence of different seasonal patterns for malaria. In a 3-year study in Roraima, 8 municipalities showed increased risk for malaria during the middle of the dry season or shortly after the wet season (*9*). Other literature on seasonal patterns is limited to local and short (<3 years) longitudinal studies that lack statistical analysis. Although different seasonal patterns emerge in graphs, the collage of different data sources makes formulating a cohesive picture of these patterns in the Amazon region difficult.

At the regional level, interannual climatologic cycles provide insight into low-frequency malaria patterns. In Columbia, El Niño events (caused by warming sea surface temperatures in the central tropical Pacific) are associated with warmer temperatures, higher dew points, and less precipitation and river discharge. These climate changes have been associated with increases in malaria during the second half of El Niño years and during the following year (*10*). Similarly, malaria incidence has increased during the year after an El Niño event in Venezuela and Guyana (*11*).

Using monthly reports of malaria and precipitation from across the Brazilian Amazon Basin, we demonstrate that malaria incidence and precipitation patterns vary throughout this large region and are influenced by the extent of wetlands.

## The Study

**Figure 1:**
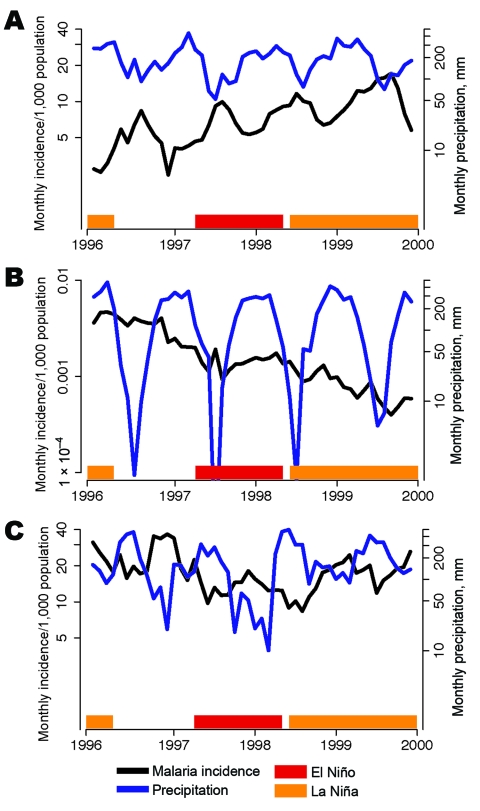
Malaria incidence per 1,000 population (black lines) and mean monthly precipitation (blue lines) during La Niña (orange bars) and El Niño (red bars) events for the states of A) Amazonas, B) Mato Grosso, and C) Roraima.

**Figure 2:**
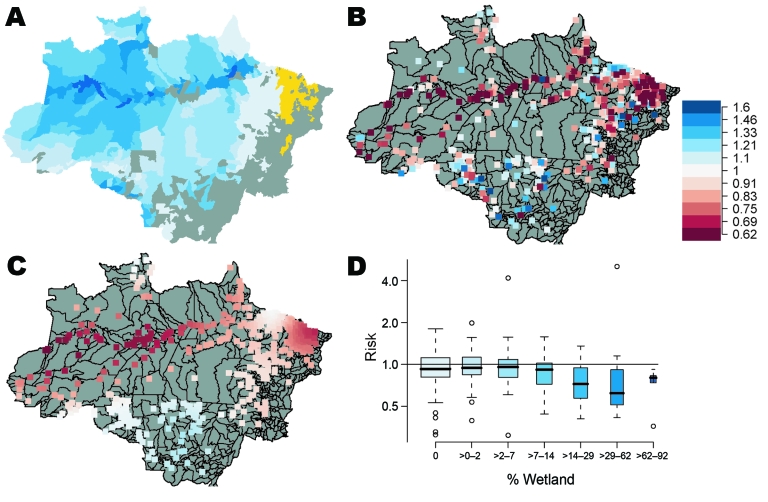
Connection of malaria incidence and precipitation risk ratios to wetlands. A) Percentage of wetlands in Amazon Basin counties (shades of blue), counties without wetlands data (yellow), and counties with <80 total malaria cases (gray). Wetland colors correspond to percentage wetland values in panel D. B) Risk ratios for malaria incidence for 1 SD (≈14 cm) change in monthly precipitation (January 1996–December 1999), plotted at each county seat of government; C) spatially smoothed risk ratios for ≈14-cm changes in monthly precipitation. In both panels, red shaded squares show reduced risk for ≈14-cm increase in monthly precipitation; blue shaded squares show increased risk for malaria with increased precipitation. D) Boxplot of risk ratios for malaria incidence for ≈14-cm changes in monthly precipitation, by percentage wetland cover. Box width is proportional to the number of counties in each box. Error bars indicate interquartile ranges, and thick horizontal bars indicate the median.

We used monthly reports of slide-confirmed malaria and annual census–based population data from 434 counties (municípios) in the Brazilian Amazon region for 1996–1999, during which no coordinated national malaria interventions occurred (*12*). To study the relationship of reported malaria cases to climate, we used monthly precipitation and temperature from the CRU TS 2.1 gridded climate data set for selected states (*13*) (Figure 1). To consider how the precipitation–malaria relationship depends on surface water conditions, including the extent of open water and wetlands, we used 100 m × 100 m maps from the JERS-1 Synthetic Aperture Radar satellite and calculated the percentage of maximum inundatable open water and wetland coverage for each county (Figure 2, panel A) (*14*). In this region, monthly temperatures were between 24.6°C and 29.4°C (well within the range for optimal malaria transmission) for 95% of the observations (18,416 of 19,364) included in the analysis (temperature relationships not shown).

Evaluation of seasonal patterns requires comparability of the models across regions. If the lag and the rainfall coefficient vary across regions, meaningful geographic comparisons would be difficult to achieve because neither the lag nor coefficient have consistent meanings across models. To interpret results, either the coefficient must be fixed and the lags varied (difficult to do) or the lags must be fixed and the coefficients varied (relatively easy to do). The aim is to describe the variable patterns of malaria incidence and precipitation, not create a highly predictive model. We chose to fix the lag and vary the coefficients.

To assess the association between malaria incidence and precipitation data, we estimated the rate ratio of malaria incidence associated with 1 SD-increase in monthly precipitation (≈14 cm) for each county by using the following Poisson regression model, which includes a flexible temporal trend represented as a natural cubic spline with 6 degrees of freedom (Figure 2, panel B):

malaria*_it_* ≈ Poisson(μ*_it_*)

log μ*_it_* = log pop*_it_* + α*_i_* + β*_i_*precip*_it_* + *f_i_*(*t*)

β*_i_* ≈ Normal(*g*(lat*_i_*, lon*_i_*), σ^2^)

The (estimated) regression coefficients from the county-specific models were then modeled as a spatially smooth surface, a thin-plate spline. Degrees of freedom for the thin-plate spline were selected using generalized cross-validation (Figure 2, panel C).

The relationships between precipitation and malaria incidence in the Amazon Basin are spatially varied and change signs, depending on the region. Positive correlations between monthly precipitation and malaria incidence (rate ratios >1) occur in the upland regions of the southwest and central Amazon Basin, whereas negative correlations between precipitation and incidence (rate ratios <1) occur in the north, largely along the main waterways of the Amazon River and the major wetland regions of the Basin (Figure 2). For a ≈14-cm increase in monthly rainfall, the malaria rate can double in the upland area, yet decrease by up to 80% along the main Amazon channel. The p values of the precipitation coefficient are 0.0002–0.0009 along the main waterways and 0.004–0.10 in uplands areas.

We hypothesize that this reversal of the malaria–precipitation relationship from positive to negative is related to the extent of open water and wetlands in the Basin. Mosquito habitats in wetlands or along large rivers may wash out or become too deep during months with high precipitation, but in areas with fewer wetlands, mosquito habitats are limited by precipitation.

To test this hypothesis, we compared the malaria–precipitation association for 338 counties that reported >80 cases of malaria over the 48 months against the estimated percentage of open water and wetland cover for each county (Figure 2, panel D). As expected, the precipitation-linked risk for malaria fell as the percentage of wetland in each county increased, but the risk for malaria varied in counties with low percentages of wetlands. The central-east region had the lowest level of malaria incidence, which may explain why this region also lacked a malaria-precipitation relationship.

## Conclusions

Explanations similar to our wetlands hypothesis have been reported. Studies have proposed that flooding created new pools of water suitable for mosquito larvae as the water levels slowly receded from alluvial forests along the Rio Branco River in Roraima and the Maroni River on the frontiers of Suriname and French Guiana (*6*,*15*). Our results suggest that monthly precipitation along the Amazon Basin can have both strong positive and negative associations with malaria incidence.

Further research is needed to address the limitations of our study, including its short time frame and the crude countywide approximation of percentage wetlands as an exposure. The quality and reliability of the health data were concerns, but we verified that the distribution of null reporting was unbiased temporally and spatially. Also, our study did not quantify increasing malaria incidence in response to increasing or decreasing precipitation or the impact of lag factors. Instead, we focused on the seasonality of these patterns until longer data series of malaria incidence and climate data are available.

Our evidence suggests that precipitation drives malaria risk in the Amazon Basin, but the relationship varies in the uplands (more precipitation, more/less malaria) and is negative in areas dominated by wetlands and large rivers (more precipitation, less malaria). Our findings show the need to account for local landscape characteristics, especially the extent of wetlands and open water, in regional to global projections of the effects of climate change on malaria. Better understanding the impact of climate and landscape on malaria will improve our ability to assess health risks.
